# Prognostic Value of Spatial and Topological Features of Tumor Microenvironment in Classic Hodgkin Lymphoma

**DOI:** 10.32604/or.2026.079403

**Published:** 2026-06-16

**Authors:** Irene Bernal-Florindo, Jose Angel Raposo-Puglia, Felix A. Ruiz, Jose Perez-Requena, Cristian Benavides-de la Fuente, Javier Galan, Maria Jose Berruezo-Salazar, Marcial Garcia-Rojo, Cecilia Fernandez-Ponce, Antonio Santisteban-Espejo

**Affiliations:** 1 Department of Pathology, Jerez de la Frontera University Hospital, Jerez de la Frontera, Cadiz, Spain; 2 Biomedical Research and Innovation Institute of Cadiz (INiBICA), Research Unit, Puerta del Mar University Hospital, Cadiz, Spain; 3 Department of Hematology and Hemotherapy, Puerta del Mar University Hospital, Cadiz, Spain; 4 Department of Biomedicine, Biotechnology and Public Health, Faculty of Medicine, University of Cadiz, Cadiz, Spain; 5 Department of Pathology, Puerta del Mar University Hospital, Cadiz, Spain; 6 Department of Immunology, Puerta del Mar University Hospital, Cadiz, Spain; 7 Department of Hematology, Jerez de la Frontera University Hospital, Jerez de la Frontera, Spain; 8 Department of Medicine and Surgery, Faculty of Medicine, University of Cadiz, Cadiz, Spain

**Keywords:** Classic Hodgkin lymphoma, tumor microenvironment, computational pathology, eco-oncology, spatial biology

## Abstract

Classic Hodgkin lymphoma (CHL) constitutes a B-cell malignant lymphoid neoplasm derived from the germinal center. Despite current treatment protocols based on chemotherapy, radiotherapy, anti-cluster of differentiation (CD) 30 antibody-drug conjugates, immunotherapy, and hematopoietic stem cell transplantation (HSCT), between 10% and 20% of CHL patients fail to achieve a complete response. The reasons underlying this lack of treatment sensitivity remain unclear. Traditionally, clinical and analytical variables have constituted the cornerstone of CHL prognostic model development. However, in recent years, the distribution and spatial relationships of cancer and immune cells within the CHL tumor microenvironment (TME) have emerged as novel potential candidates for risk stratification and treatment personalization. Underpinning this field of research, advances in digital image analysis (DIA) and computational pathology (CP) tools have been fundamental, as these methods enable objective quantification of TME elements and the definition of their topological arrangement. Novel CHL prognostic models integrating data across DNA sequencing in peripheral blood (liquid biopsy), single-cell RNA sequencing (scRNAseq), spatial transcriptomics, positron emission tomography/computed tomography (PET/CT) imaging, and topological features of TME could inform better clinical decision-making in the near future. In this work, we review the current state of CP and DIA studies in CHL, emphasizing the transition from traditional histopathological characterization to computational biology, highlighting the prognostic value of TME components, and proposing an updated framework for CHL tumor evolution and cellular dynamics as ecological systems. This study aims to review the contributions of DIA and CP in clinical and translational research on CHL. The results of this study may contribute to the identification of new prognostic biomarkers and their use in both the design of risk stratification models and clinical trials for CHL.

## 1 Introduction

### 1.1 Classic Hodgkin Lymphoma: a Model of Translational Research

Classic Hodgkin lymphoma (CHL) is a B-cell neoplasm differentially categorized according to the 5th Edition of the World Health Organization Classification of Haematolymphoid Tumours (WHO-HAEM5) [1] and the International Consensus Classification (ICC) [2]. The term “nodular lymphocyte predominant Hodgkin lymphoma” (NLPHL) is listed under the family of Hodgkin lymphomas in the latest edition of the WHO classification; however, a new entity, called “Nodular lymphocyte predominant B-cell lymphoma” (NLPBL), was proposed by the ICC based on major biological and clinical differences from CHL. This review focuses on CHL, which is clearly differentiated from NLPHL and NLPBL in molecular, histopathological, clinical, and therapeutic terms.

Since its first description in 1832 by Thomas Hodgkin [3] and its cytological and histopathological characterization by Carl Sternberg [4] and Dorothy Reed [5], the history of CHL constitutes a model of successful collaboration between basic and clinical research, which has led to the gradual achievement of fundamental clinical objectives [6]. Currently, the cure rate of CHL is near 90%, being a paradigm of curable cancer.

Several milestones in this translational research model in oncohematology deserve special mention. Among them are the recognition of the B-cell origin in CHL as described in two seminal studies by Küppers et al. [7,8], as well as the progressive optimization of chemotherapy [9,10,11,12,13] and radiotherapy [14,15,16] regimens throughout the twentieth century, the improvement of conditioning and supportive care [17,18,19,20,21] and management of hematopoietic stem cell transplantation (HSCT) [22,23] and the development of targeted therapies against the cluster of differentiation (CD) 30 antigen [24,25,26] and the Programmed Cell Death protein-1 (PD-1) [27] which has significantly improved the management of refractory/relapsed CHL (R/R CHL). In parallel, the de-escalation of treatment intensity based on improved assessment of therapeutic response using 18-fluorodeoxyglucose ([18F]FDG) positron emission tomography combined with computed tomography (^[18F]FDG^PET/CT) [28,29,30,31], the development of more accurate [32,33,34,35] and robust [36,37] clinical prognostic models and a better comprehension of the key biological features of the disease [38,39] have directed CHL research toward its current challenges.

### 1.2 New Risk Stratification Models for Classical Hodgkin Lymphoma: the Role of Liquid Biopsy, Metabolic Imaging, and Computational Pathology

Despite the progress made, there are still 10%–20% of patients diagnosed with CHL who will die due to the progression of the disease. Thus, the design of strategies to enable early identification of this high-risk subset of CHL patients constitutes an essential goal in the current era. Three technologies could support the development of new prognostic models that, eventually, will capture in the future the biological complexity of high-risk CHL: liquid biopsy, metabolic evaluation through artificial intelligence (AI) assisted ^[18F]FDG^PET/CT imaging, and spatially resolved analyses. The integration of these approaches into multimodal prognostic tools is expected to enable the identification of aggressive disease features both at diagnosis and during disease monitoring.

First, given that circulating tumor DNA (ctDNA) fragments are detectable in the blood of CHL patients at concentrations sufficient for extraction and sequencing (~30 ng/mL) [40], liquid biopsy will be increasingly adopted as a precise genotyping and disease-monitoring strategy. Previous studies have shown that allele frequencies found in the blood of CHL patients are higher than those detected in lymph node samples (1.99% vs. 1.6%, *p* = 0.02) [41]. Moreover, a longitudinal study of ctDNA concentration has shown correlation with the degree of treatment response and clonal evolution [42]. Furthermore, ctDNA concentrations are lower in patients who achieve remission compared to those who do not (*p* < 0.001, at a 96 months of follow-up) [43]. Liquid biopsy in CHL, therefore, allows the identification of mutations in peripheral blood that cannot be detected through tissue-based analyses and facilitates the identification of patients at risk of treatment failure, who may benefit from more intensive, dynamic, or molecular-guided therapy strategies [44,45].

Second, application of AI algorithms in PET/CT evaluation constitutes an active field of research with potential advantages in the management of analysis time, interobserver variability, and response prediction. On the one hand, evaluating PET/CT images in patients with CHL is a time-consuming and resource-intensive process. The complexity of analyzing these images can stem from the need to differentiate between tumor processes and inflammatory or infectious processes, which are common in this immunocompromised population. AI-based software for analyzing medical images using PET/CT could reduce analysis time by automating some processes and requesting a medical professional review only in particularly difficult cases. On the other hand, research areas such as interobserver variability and response prediction using AI algorithms have been examined in recent literature [46]. Particularly, in CHL, automatic segmentation and calculation of PET/CT parameters using AI tools have been compared with traditional methods. In a recent study [47], no statistically significant differences were observed for parameters used in clinical routine such as the Standardized Uptake Value maximum (SUV max) and Metabolic Tumor Volume (MTV), at either the interim or end-of-treatment assessment in a cohort of 32 CHL patients. These data suggest that the introduction of AI tools in the analysis of PET/CT medical images in patients diagnosed with CHL will be gradual in the near future, but still require standardization and normalization of the tools used.

However, novel AI algorithms are not yet standardized in the clinical setting; as a consequence, other studies have shown that AI algorithms tend to overestimate PET/CT parameters (e.g., the Deauville score) [48,49]. These “false positive” evaluations of AI methods in the analysis of PET/CT images raise the concern of unnecessary treatment escalation and higher toxicity. Thus, harmonizing the AI algorithms used for PET/CT image analysis in CHL constitutes an important clinical challenge.

Third, CHL tissue biopsies are characterized by a scarce tumoral fraction (1%–10%), termed Reed-Sternberg cells and mononuclear (Hodgkin), lacunar, and mummified variants, surrounded by a prominent tumor microenvironment (TME). The TME of CHL consists of a heterogeneous tissue space composed of T lymphocytes, B lymphocytes, macrophages, neutrophils, eosinophils, and histiocytes, among other minority cell types [50]. Traditionally, the TME of CHL has been considered the result of an impaired immune system unable to control tumor progression effectively, a perspective aligned with the “reductionist view” described by Hanahan and Weinberg in their landmark 2000 paper [51].

Nevertheless, TME of CHL constitutes a precise network of cellular, molecular, and spatially defined interactions orchestrated to enhance tumor progression and treatment resistance. The spatial localization of cellular elements, the interactions between cancerous and “non-cancerous” cell populations, and their genomic and molecular evolution in CHL are the consequence of dynamic evolutionary processes. In this regard, the discipline of eco-oncology [52] and the study of CHL from an ecological perspective can serve as a basis for a new approach to monitoring and treatment strategies for CHL. Recent evidence from digital image analysis (DIA) and computational pathology (CP) tools demonstrates that the histological architecture of CHL reflects a precise spatial topology created by cancer cells throughout its biological evolution [53,54]. The guiding role of ecological principles in cancer biology has been demonstrated by the fact that the hallmarks of cancer are spatially defined, shaping both cancer evolution and drug sensitivity [55].

In this review, we focus on the clinical and prognostic significance of spatial and topological features of TME in CHL, emphasizing the need to bring together the fields of molecular biology, DIA, CP, biomathematics, and AI computing to generate new, more accurate, and clinically relevant prognostic models in CHL. The aim of this study is to review the contributions of DIA and CP in clinical and translational research on CHL.

## 2 From Histopathological Characterization to Computational Biology in Classic Hodgkin Lymphoma

### 2.1 Pioneering Studies on the Application of Digital Image Analysis to Classical Hodgkin Lymphoma

There is a growing body of knowledge on the applications of DIA and CP for identifying prognostic biomarkers in TME of CHL. In this milieu, pioneering studies reported interesting results, leading to a transition from traditional studies focused on CHL histopathology [56,57,58] to the novel approaches centered on slide digitization and application of DIA algorithms. In this process, scanning and feature extraction with CP algorithms constitute an opportunity to develop a real TME-based risk stratification model. This aspect constitutes a key objective in CHL research, as a fraction (10%–20%) of patients are still primarily refractory or relapse (R/R) after achieving complete metabolic response (CMR) with current chemotherapy and radiotherapy regimens [59].

**Figure 1:**
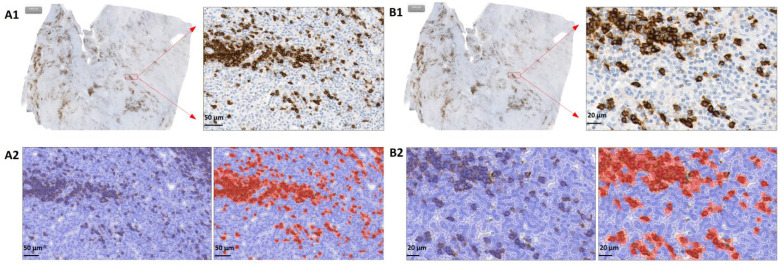
Cellular segmentation and quantification of CD30^+^ cells in CHL using QuPath. (**A1**) Visualization of a digital slide of a whole lymph node sample (10×). (**A2**) Cell segmentation/detection (left image): The algorithm recognizes and segments each cell (blue color) (10×). CD30 quantification (right image): The algorithm quantifies CD30-positive cells (red color) (10×). (**B1**) Visualization of a digital slide of a whole lymph node sample (20×). (**B2**) Cell segmentation/detection (left image): the algorithm recognizes and segments each cell (blue color) (20×). CD30 quantification (right image): the algorithm quantifies CD30-positive cells (red color) (20×).

In 2013, the first study linking CHL and CP was published [60]; the authors described a workflow for exploiting CD30-stained lymphatic tissue image data in CHL samples. Later, in 2016, mathematical studies by Watts and Strogatz on small-world networks [61] and by Barabási and Albert on random networks [62] enabled the construction of a cellular graph from CHL histopathological slides [63]. This cell graph of CD30^+^ tumoral (Hodgkin/Reed-Sternberg or HRS) cells is defined by two properties: 1) greater degree than a random model, i.e., the location of tumoral cells in CHL is not spatially random, but rather there is a certain degree of clustering between cells; and 2) absence of freedom of scale, i.e., there are no highly connected groups of HRS cells (defined as hubs) and a distant “disconnected” tumoral population, but rather a connection described by Watts and Strogratz as “small-world networks”. Fig. 1 (own data) shows the process of scanning, cell identification, and quantification of CD30^+^ tumoral HRS cells in CHL using the software commonly employed by our research group (MembraneQuant & NuclearQuant, QuantCenter Software, 3DHistech Ltd., Budapest, Hungary, and QuPath, version 0.4). Fig. 1 shows the cellular segmentation and quantification of CD30^+^ cells in CHL using QuPath (version 0.4).

These studies have certain limitations, such as evaluating a single cell population (CD30^+^ tumor cellularity), omitting the other cellular components that integrate the TME (T lymphocytes, B lymphocytes, plasma cells, dendritic cells, eosinophils, macrophages, among others). In 2020, Hanning et al. demonstrated the preference of tumor cells for certain neighboring cells based on their morphology [64], and recently, novel parameters such as cell velocity, number of cell contacts, and cell contact time have been obtained and evaluated in CHL preparations visualized in 3D and 4D images [65,66,67].

### 2.2 Prognostic Stratification in Classic Hodgkin Lymphoma Using Digital Image Analysis and Computational Pathology

The aforementioned studies, however, do not examine the impact of DIA data on treatment response. Consequently, prognostic information on the evolution of CHL patients cannot be extracted. For this purpose, the first paper was published in 2021 by members of the German Hodgkin Study Group (GHSG) [68]. In this analysis, using whole slide imaging (WSI) of histopathological CHL samples from 487 patients, a low B-cell content in the TME (<21%) was identified as an adverse prognostic factor. This finding was reported in advanced-stage CHL patients who received Bleomycin, Etoposide, Adriamycin, Cyclophosphamide, Vincristine, Procarbazine, and Prednisone (BEACOPP) chemotherapy as first-line treatment.

After the GHSG research, our group reported that a percentage of 2% of HRS cells, quantified using WSI and DIA methods, statistically significantly discriminated patients achieving CMR from those R/R patients following first line treatment with Adriamycin, Bleomycin, Vinblastine and Dacarbazine (ABVD), both for overall survival (OS) (*p* = 0.001) and progression-free survival (PFS) (*p* = 0.005) [69]. Furthermore, CHL patients with a T cell percentage below 26.70% in the TME showed a statistically significantly shorter OS (*p* = 0.019) and PFS (*p* = 0.041). In addition, the combined impact of HRS and T cells percentages identified a high-risk subgroup of CHL. No prognostic impact of B cells was identified in this study, probably due to differences in cohort sizes (*n* = 85 vs. *n* = 487), distribution of disease stages (localised, intermediate, and advanced CHL vs. advanced CHL), and chemotherapy regimens (ABVD vs. BEACOPP).

Moreover, the immune response mediated by Programmed Cell Death Protein-1 (PD-1)/Programmed Cell Death Ligand-1 (PD-L1) also appears to play a prognostic role in CHL. Using the Transparent Reporting of a Multivariable Prediction Model for Individual Prognosis or Diagnosis (TRIPOD) guidelines [70], PD-L1/CD30 ratio identified a group of patients with an increased risk of failure to ABVD chemotherapy as first-line therapy: CHL patients with PD-L1/CD30 ratio > 47.1 showed a lower OS (median OS: 53.7 months; 95% confidence interval [CI]: 28.7 to 78.7) compared to those below this threshold (median OS: 105.4 months; 95% CI: 89.6 to 121.3) (*p* = 0.04). When adjusted for covariates, PD-L1/CD30 ratio retained its prognostic impact for both OS (Hazard Ratio [HR]: 1.005; 95% CI: 1.002 to 1.008; *p* < 0.001) and PFS (HR: 3.442; 95% CI: 1.045 to 11.340; *p* = 0.04) based on data from three different centers.

Recently, GHSG reassessed the prognostic impact of B lymphocytes in CHL using DIA technologies [71]. On this occasion, a subgroup of patients was treated with ABVD chemotherapy (4 cycles) or ABVD and escalated BEACOPP (BEACOPPe) (defined as “2 × 2” regimen), which modified the homogeneity of the treatments included. Using a cutoff value of 8%, determined by Receiver Operating Characteristics (ROC) curves, CHL patients with ≤8% B lymphocytes in the TME showed a lower 5-year PFS (71% vs. 83%). However, these differences were not statistically significant (*p* = 0.08) and were not reflected in the OS data (*p* = 0.34).

**Figure 2:**
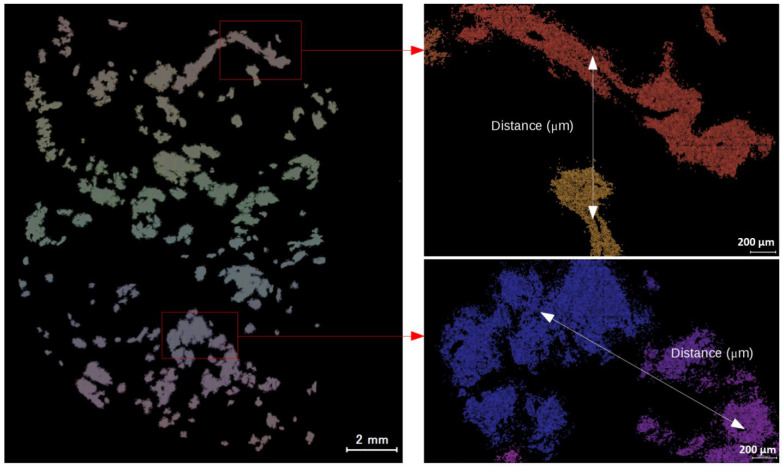
Spatial visualization and distance mapping of CD21^+^ FDC network within a CHL lymph node sample generated using the Napari software (version 0.6.6). Each positive cell cluster is distinctly color-coded to facilitate the identification of topological patterns.

Moreover, the combined use of WSI and next-generation sequencing (NGS) in CHL allows assessment of the combined impact of genomic and histopathological variables. In 2025, we reported [72] data on the quantification of B lymphocytes in the TME of 220 diagnostic slides from 110 CHL patients, and on the sequencing of tissue biopsies using a targeted panel that included 47 genes recurrently mutated in mature B-cell neoplasms. A combined reduction in B cell content (<8%) and the absence of mutations in apoptosis-associated genes (*ABL1*, *BIRC3*, *CASP8*, and *FAS*) were identified as adverse prognostic factors. CHL patients exhibiting this combined event (low B cells in TME and absence of mutations in apoptosis genes) presented a significantly lower OS (median OS: 31.5 months, 95% CI: 0 to 69.7 months) compared with patients without this feature (median OS: 84.7 months, 95% CI: 61.9 to 107.5 months) (*p* = 0.01). Additionally, this subgroup showed significantly lower PFS (median PFS: 8.5 months; 95% CI: 7.5 to 9.5 months) compared to CHL enriched in B cells or with mutations in genes linked to apoptosis (median PFS: 55.2 months; 95% CI: 42.4 to 68 months) (*p* < 0.001). Fig. 2 (own data) shows the process of scanning, cell identification, and quantification of CD21^+^ cells in TME of CHL using the software MembraneQuant & NuclearQuant (QuantCenter Software, 3DHistech Ltd., Budapest, Hungary, and QuPath, version 0.4). Fig. 2 illustrates the calculation of distances within the network of CD21^+^ follicular dendritic cells (FDC) in a diagnostic CHL lymph node sample using the Napari software (version 0.6.6). This spatial characterization is critical for defining the specialized niches and cellular distances that modulate the interaction between HRS cells and their surrounding microenvironment.

## 3 Deciphering the Prognostic Value of TME Components in Classic Hodgkin Lymphoma

### 3.1 Current Risk Stratification Models in Classical Hodgkin Lymphoma: from the International Prognostic Score to the Advanced-Stage Classic Hodgkin Lymphoma International Prognostic Index

Currently, there are six major prognostic stratification models validated for its routine application in the clinical management of CHL patients: the model proposed by GHSG [73], the model of the European Organisation for Research and Treatment of Cancer (EORTC) [74], the National Cancer Institute of Canada (NCI-C) model [74], the National Comprehensive Cancer Network (NCCN) model [75], the International Prognostic Score (IPS), designed by Hasenclever and Diehl [32], and the Advanced-stage CHL International Prognostic Index (A-HIPI) [76], designed by the HoLISTIC Consortium (Hodgkin Lymphoma International Study for Individual Care).

The first four models (GHSG, EORTC, NCI-C, and NCCN) are used in patients with localized disease (stages I–II). In contrast, the latter two (IPS and A-HIPI) are used in patients with advanced disease, defined as stage II with B symptoms (fever, profuse night sweats, or weight loss of more than 10% in the last six months) accompanied by bulky masses or extranodal involvement, as well as stages III–IV. Table 1 describes the prognostic variables considered in each of the models currently used at the CHL for treatment strategy planning.

**Table 1:** Prognostic stratification models in classic Hodgkin lymphoma.

Prognostic Model	Risk Factors	Clinical Application
**EORTC**	- Bulky mediastinal mass > 1/3 thoracic diameter. - Age ≥ 50 years. - Elevated erythrocyte sedimentation rate (≥30 with B symptoms or ≥50 without B symptoms). - Involvement of 4 or more lymph node regions.	- Favorable: Supradiaphragmatic stages I–II without risk factors. - Unfavorable: supradiaphragmatic stages I–II with ≥1 risk factors.
**GHSG**	- Bulky mediastinal mass. - Extranodal involvement. - Elevated erythrocyte sedimentation rate (≥30 with B symptoms or ≥50 without B symptoms). - Involvement of 3 or more lymph node regions.	- Favorable: stages I–II without risk factors. - Unfavorable: Stages I or IIA with ≥1 risk factors, or Stages IIB with (C) or (D) but without (A) and (B).
**NCI-C**	- Histology other than lymphocytic predominance/nodular sclerosis. - Age ≥ 40 years. - Erythrocyte sedimentation rate ≥ 50. - Involvement of 4 or more lymph node regions.	- Favorable: stages I–II without risk factors. - Unfavorable: stages I–II with ≥ 1 risk factors.
**NCCN**	- Large mediastinal mass > 1/3 of the chest diameter or any mass > 10 cm in diameter. - Erythrocyte sedimentation rate ≥ 50 or any B symptom. - Involvement of 3 or more lymph node areas. - >1 extranodal involvement.	- Favorable: stages I–II without risk factors. - Unfavorable: Stages I and II with ≥ 1 risk factors (differentiating between bulky disease and other risk factors).
**IPS**	- Male sex. - Age ≥ 45 years. - Stage IV. - Albumin < 4 g/dL. - Hemoglobin < 10.5 g/dL. - Leukocytosis ≥ 1.5 × 10^10^/L. - Lymphopenia < 6 × 10^8^/L or <8% of white blood cell count.	- The addition of risk factors decreases OS at 5 years; specifically, patients with an IPS ≥ 3 are considered high-risk patients (5-year OS < 59%).
**A-HIPI**	- Age. - Sex. - Stage. - Bulk. - Absolute lymphocyte count. - Hemoglobin. - Albumin. - Statistical treatment for continuous variables revealed nonlinear relationships with outcomes (5-year OS, 5-year PFS) for absolute lymphocyte count and age.	- Superior discrimination for 5-year OS compared with the IPS. - Overestimation in the highest deciles of predicted risk in the A-HIPI. - Improved calibration for both 5-year PFS and 5-year OS compared with IPS.

Note: EORTC, European Organisation for Research and Treatment of Cancer; GHSG, German Hodgkin Study Group; NCI-C, National Cancer Institute of Canada; NCCN, National Comprehensive Cancer Network; IPS, International Prognostic Score; A-HIPI: Advanced-stage CHL International Prognostic Index; OS, Overall survival; PFS, Progression-Free Survival.

### 3.2 Prognostic Models Based on the Assessment of the Tumor Microenvironment in Classical Hodgkin Lymphoma

Risk stratification of CHL in the clinical setting constitutes a critical step, as it is required for planning the optimal therapeutic strategy (most effective and least toxic). In particular, risk categorization is needed to distinguish between patients with localized and advanced disease. Patients with localized stages should then be subclassified into favorable (I–II without risk factors) and unfavorable (I–II with risk factors or III–IV). The intensity of chemotherapy and radiotherapy schedules to be administered during the course of the treatment varies among the different subgroups.

In patients with advanced-stage disease (IIB, III–IV), incorporating IPS variables decreases 5-year OS. Specifically, CHL patients with an IPS of three or higher are classified as high risk with a 5-year OS below 59%. In addition, it should be noted that, because of its recent communication using data from >5000 CHL patients with newly diagnosed advanced-stage CHL and the widespread use expected in the coming years, the A-HIPI model has improved outcome predictions using 5-year OS and 5-year PFS as clinical endpoints compared to the traditional IPS [76].

However, none of the above models incorporate histopathological, cytogenetic, or molecular variables, as they are based exclusively on clinical, analytical, and disease extension parameters. This fact is striking in clinical research on CHL, given the progress made in understanding the molecular basis of the disease over the last 20 years. The clinical translation of preclinical results is complex, and in most cases the prognostic biomarkers that have been proposed as clinically actionable have not demonstrated external validity.

In this context, the study of the cell populations that comprise the TME is one of the most developed areas of translational research in CHL. On the one hand, the scarcity of tumor cellularity and the prominence of different TME cell populations suggest a key role for TME in the pathogenesis and natural history of the disease. Different cell types have been evaluated in recent years. Firstly, the T cell population constitutes the most abundant cell type in the CHL TME. The presence of regulatory T cells within the tumor-reactive microenvironment could explain the suppression of the antitumor immune response observed in these patients. In a previous study [77], a low infiltration of regulatory T cells (FOXP3^+^) and a high proportion of cytotoxic T lymphocytes (TIA1 and granzyme B) were associated with decreased survival. Greaves et al. [78] validated the combination of FOXP3 and CD68 as biomarkers for establishing prognostic stratification groups.

On the other hand, the work by Alonso-Álvarez et al. [79] demonstrated that in diagnostic biopsies of patients with CHL, a high proportion of CD8^+^ T lymphocytes (≥15%) and CD4^+^ T lymphocytes (≥75%) correlated with longer treatment failure-free survival (FFS) at 10 years. Of note, natural killer (NK) cell infiltration and activation in TME appear to confer a favorable prognosis in CHL [80]. Álvaro-naranjo et al. [81] reported that a low number of activated NK cells is associated with traditionally adverse prognostic factors, including the presence of B symptoms at diagnosis and the advanced disease stages. However, NK cell infiltration in the TME of CHL remains largely diminished [82], and their precise role in eliminating HRS cells remains an active field of research.

Regarding the study of the mononuclear phagocytic system (MPS) and its cellular components, the pioneering work of Steidl et al. [83] reported a negative prognostic impact for the increased CD68^+^ macrophages, both after first-line treatment (*p* = 0.03 for PFS; *p* = 0.003 for disease-specific survival, DSS) and in response to HSCT (*p* = 0.008), improving the predictive capacity of the IPS (*p* = 0.003 vs. *p* = 0.03). These data have been validated in subsequent studies [84], suggesting an adverse prognostic role for high levels of CD68^+^ cells in CHL.

Dendritic cells, follicular dendritic cells, and neuroendocrine cells, however, have been relatively understudied in CHL. On the one hand, an association has been reported between low levels of circulating dendritic cells [85] and survival in patients with CHL. Furthermore, employing immunohistochemistry (IHC), the reduction of CD21^+^ FDC in biopsy specimens of CHL, and the degree of destruction of the FDC network, correlated with a lower survival in CHL [86,87].

To the best of our knowledge, only one study [88] has reported aberrant expression of synaptophysin (a neuroendocrine marker) in CHL. In this study, survival analysis was not conducted. However, it is well established that the neuroendocrine axis modulates immune system activity and is expressed in other tumors as a mechanism of resistance to treatment [89,90]. Further studies are needed to evaluate the expression of neuroendocrine markers in CHL and their association with treatment responses.

## 4 Tumor Evolution and Cellular Dynamics in Classic Hodgkin Lymphoma As Ecological Systems

In a groundbreaking publication, Reynolds et al. [52] posed a thought-provoking question: “Why should oncologists think like ecologists?” The histopathological architecture, molecular evolution, and response to treatment of tumors can be viewed as evolutionary processes. Several basic principles of cancer biology, in fact, mirror ecological notions: i.e., tumor growth dynamics can be analyzed using the logistic population growth model, in which the proliferation rate of cancer cells decreases as the carrying capacity of the environment (i.e., the anatomical location of the tumor) decreases. Moreover, clonal evolution from a common mutated ancestor and divergence under external pressure (chemotherapy, radiotherapy, nutrient deprivation, spatial anatomical limitations) are essentially the results of changes over time in heritable traits. Consequently, genetic variability, competition, and adaptation within an environment of limited biological resources and the survival of the fittest, are Darwinian concepts that have motivated, in recent years, the study of cancer in light of the theory of natural selection.

Due to its unique histopathological characteristics, knowledge and application of the principles of eco-oncology may be particularly useful in CHL. As previously reported, the TME cell populations in CHL exceed the tumoral compartment. Thus, interactions among tumoral HRS cells, stromal cells, and immune system cells, together with the formation of immunoprivileged niches and the emergence of spatial topologies that favor tumor growth and drug resistance, have been identified as distinctive features in various solid tumors [55], and particularly in CHL.

In the current contribution of CP and DIA to the biological knowledge of the TME of CHL, the definition of the features of the immunoprivileged niches around HRS cells constitutes the focus of active research. In this sense, identifying cells in proximity to HRS cells after digitalization of histopathological CHL slides has allowed the study of secreted or cell-surface ligands expressed by microenvironmental cells within these tumoral niches. Shanmugam et al. [91] described a spatially aware ligand-receptor model in CHL. They identified IL13 as a central survival factor for HRS cells, providing a biological basis for testing IL13-directed therapies in CHL. In 2017, Carey et al. [53] demonstrated a close relationship between HRS cells and PD-L1^+^ macrophages. In particular, the HRS cells release a set of cytokines that attract PD-L1^+^ macrophages to their vicinity. This colocalization facilitates immune evasion through the PD-L1/PD-1 axis. In these samples, the cellular density of PD-L1^+^ macrophages and PD-L1^+^ CD4^+^ T cells in the vicinity of the HRS cells was higher than the density far from the tumoral compartment. This immunoprivileged niche protects HRS cells from pharmacological actions. Overall, these data demonstrate that HRS cells create a unique topology that favors resistance to PD-L1 blockade and imply CD4^+^ T cells as a pharmacological target for drugs such as nivolumab and pembrolizumab.

During cancer treatment, pharmacological agents inhibit cell growth and simultaneously select the best adapted-clones; this clonal selection phenomenon, in parallel with clonal drift (extinction of non-adapted clones), drives cancer evolution. Nevertheless, the divergence from the common mutated ancestor and the selected mutations differed depending on the treatment [92]. In CHL, clonal divergence, a key principle of variation in ecological systems, has been demonstrated by analyzing the evolution patterns of longitudinal ctDNA samples. In this regard, Spina et al. [42] reported differential clonal shift patterns under chemotherapy, brentuximab vedotin and immunotherapy. In particular, patients in the immunotherapy group (*n* = 5) were treated with anti-PD1 drugs, specifically Nivolumab. In CHL patients treated with chemotherapy (ABVD) or brentuximab vedotin, mutations inferred from the ancestral clones (STAT6, GNA13, ITPKB, and TNFAIP3) were conserved throughout the disease course, indicating preservation of early mutational events in CHL and a relative subclonal variation. However, immunotherapy courses after relapse led to the successive appearance of new clones defined by mutations absent in the mutated common ancestor (see Supplementary Fig. 9 in Spina et al. [42]). This pattern of clonal evolution after immunotherapy in CHL suggests that HRS cells respond by forming of neoantigens as a tumor-escape mechanism.

Furthermore, the complexity of the mononuclear phagocyte (MNP) network within the TME of CHL had not been thoroughly explored until the work by Stewart et al. [54]. Integrating data from single-cell RNA sequencing (scRNA-seq), spatial transcriptomics, CP, and multiplexed immunofluorescence revealed different niches in the vicinity of HRS cells. First, an inflammatory niche enriched in conventional dendritic cells (cDCs), monocytes, and macrophages. The predominance of this niche in CHL biopsies was associated with relapse and treatment failure. Classical monocytes attract and retain exhausted CD4^+^ T cells, creating an immunosuppressive cellular hub that promotes tumor surveillance. These findings are consistent with previous reports demonstrating that an increased infiltration of the TME by CD68^+^ macrophages correlates with poor outcomes in CHL [83,84]. Second, a fibrotic-stroma niche was also identified. This compartment was composed of fibroblast and endothelial cells and was associated with treatment success. The differential predominance of an inflammatory niche or a fibrotic/stroma profile in CHL could be assessed in pretreatment biopsies using CP and high-throughput analyses, and drive pathological risk-adjusted and personalized therapies.

## 5 Future Directions and Translational Applications

The application of high-throughput analysis methods such as CP, DIA, NGS, RNA-seq, spatial transcriptomics, and AI applications to the analysis of PET/CT images forms the basis of cutting-edge research in CHL. The combined evaluation of both peripheral blood and lymph node samples obtained routinely will allow for a more precise description of the molecular landscape of CHL, not statically but dynamically. Particularly, scRNA-seq and spatial analyses have recently redefined our understanding of the CHL TME. Aoki et al. identified distinct subpopulations of regulatory CD4^+^ T cells and exhausted CD8^+^ T cells that facilitate immune evasion [93]. The same research group has recently reported an enrichment of GALS9 (Galectin-9) naive B cells in early-relapsed CHL compared to newly diagnosed cases using scRNA-seq and spatial validation [94]. This study demonstrates that the naive B cell interacts with TIM3^+^ regulatory T cells, establishing a localized immunosuppressive niche that facilitates treatment resistance. The notion of “HL ecotypes” (HLE) also derives from scRNA-seq studies. Shengqin et al. [95] defined 28 unique cell states and three conserved HLEs, demonstrating that HL2 is associated with older age and Epstein-Barr virus positivity in CHL.

Furthermore, the application of concepts from population dynamics and ecology, previously well established at the macroscopic levels for species and communities, is revolutionizing our understanding of cancer as a biological phenomenon governed by the principles of natural selection. Recently, it has been shown that the hallmarks of cancer are spatially distributed and differentially clustered in the neoplastic compartment and the TME compartment of 10 different tumor types (breast, prostate, lung, brain, colorectal, ovary, bladder, liver, pancreas and kidney) [55]. These findings reshape our notion of cancer, promoting the analysis of tissue samples as ecosystems of interacting cell populations topologically favored depending on mutational profiles and neighborhood cell networks.

In the near future of CHL research, we foresee the development and clinical translation of three areas: (1) routine analysis of histopathological samples obtained at diagnosis and relapse using DIA and CP tools to define the profiles of niches accompanying HRS cells and the topologies associated with good and poor therapeutic response; (2) the progressive incorporation of liquid biopsy as a complementary tool to PET/CT in response monitoring and early detection of relapse through changes in ctDNA concentration; and (3) the development and validation of multiparametric prognostic models that include genomic, topological, and metabolic imaging information applicable in clinical practice for risk stratification.

## Data Availability

Not applicable.
